# Evaluation of serum and pleural levels tumor M2-pyruvate kinase in lung cancer patients with pleural effusion

**DOI:** 10.1186/s12890-022-02103-x

**Published:** 2022-08-10

**Authors:** Tiantian Zhang, Wei Liu, Li Li, Zou Jue, Chunhua Xu

**Affiliations:** 1 Department of Respiratory Medicine, Affiliated Nanjing Brain Hospital, Nanjing Medical University, 215 Guangzhou Road, Nanjing, 210029 Jiangsu China; 2 Clinical Center of Nanjing Respiratory Diseases and Imaging, Nanjing, 210029 Jiangsu China; 3 Department of Pathology, Affiliated Nanjing Brain Hospital, Nanjing Medical University, 215 Guangzhou Road, Nanjing, 210029 China

**Keywords:** Pleural effusion, Tumor M2-pyruvate kinase, Carcinoembryonic antigen, Lung cancer, Benign/malignant, Diagnosis

## Abstract

**Objective:**

To evaluate the diagnostic value of tumor M2-pyruvate kinase (TuM2-PK) and carcinoembryonic antigen (CEA) levels in both pleural effusion and serum in the differential diagnosis of benign and malignant pleural effusion.

**Methods:**

This prospective study was conducted among 80 patients with benign pleural effusion (BPE group) and 125 patients with malignant pleural effusion associated with lung cancer (MPE group). The levels of TuM2-PK and CEA were measured by using sandwich enzyme-linked immunosorbent assay and electrochemiluminescence. The receiver-operating characteristic curve (ROC) analysis was used to confirm the cutoff value to evaluate the diagnostic efficiency of TuM2-PK and CEA.

**Results:**

The TuM2-PK and CEA levels in pleural effusion and serum, and their ratio (P/S) were higher in MPE group than that in BPE group (*P* < 0.05). In pleural effusion and serum, the diagnostic efficiency of combined TuM2-PK and CEA for MPE was superior to either single detection.

**Conclusions:**

The combined detection of TuM2-PK and CEA has a high sensitivity for diagnosis of MPE and might provide method for rapid and accurate diagnosis of patients.

## Background

Pleural effusion is a common clinical disease, even the first symptom. There are many causes of pleural effusion, mainly tuberculosis and lung cancer in China [1]. Lung cancer is the most common malignant pleural effusion, about 20% of which is the first symptom, and 30–40% occurs in the course of the disease [2–4]. Accurate judgment of BPE and MPE is very important for the treatment of diseases, especially for patients with MPE. The delay of diagnosis means the reduction of quality of life and the shortening of survival time. The traditional pleural effusion examination has limited differential diagnosis between benign and malignant. The sensitivity of exfoliative cytology and pleural biopsy in the diagnosis of MPE was 43–95% [5]. Thoracoscopy and thoracotomy biopsy can improve the diagnostic yield, but often increase the incidence of complications [6, 7]. Therefore, the search for non-invasive identification methods with high sensitivity and specificity has attracted much clinical attention.

Pyruvate-kinase (PK) is a key enzyme in the glycolytic process that controls the generation of nucleotide triphosphate. PK has four isozymes: L-type, R-type, M1 type and M2 type. M2 type is mainly expressed in lung, distal convoluted tubules of normal kidney, embryo and or proliferative tissues. The enzyme has different tissue-specific isoforms and is a homologous tetramer in their activate state. There are many reasons for the abnormal expression of TuM2-PK in malignant tumors. In tumor cells, the isoenzyme M2-PK is converted to dimer form, overexpressed in multistep carcinogenesis and present in blood and other body fluids, possibly released from tumor cells by necrosis and cell renewal [8–10].

CEA is a glycoprotein, which mainly exists in the intestine, liver and pancreas of the fetus before 6 months of pregnancy. Due to the large molecular weight of CEA, it synthesized and released by pleural metastasis to pleural effusion is not easy to enter human blood circulation, so it rises earlier in pleural effusion and its level is also significantly higher than that in serum [11–15].

To the best of our knowledge, however, there have not been any studies on the relationship between TuM2-PK in MPE and the differential diagnosis of BPE. So this study is performed to investigate whether the expression of TuM2-PK in MPE are useful for MPE in making diagnosis.

## Methods

### Patients

205 patients who were hospitalized in Nanjing Brain Hospital and diagnosed as pleural effusion were consecutively recruited. All pleural effusion had a definite etiology, which was recorded by biochemical examination, cytology, pleural biopsy, percutaneous biopsy, endoscopy and clinical follow-up. In MPE group, 80 males and 45 females were aged (58.5 ± 11.8) years. There were 80 patients with lung adenocarcinoma (ADC), 25 patients with squamous cell carcinoma (SCC) and 20 patients with small cell lung cancer (SCLC). In BPE group, 50 males and 30 females were aged (59.2 ± 10.6) years. There were 50 patients with tuberculosis and 30 patients with pneumonia. There was no significant difference in sex and age between the two groups (*P* > 0.05).

### Diagnostic criteria for pleural effusion

According to Light's criteria, all the patients who were included in this study have a diagnosis of exudative pleural effusion. The diagnostic criteria for malignant pleural effusions were the presence of malignant tumor cells in pleural effusions or biopsy specimens. Parapneumonic effusion was diagnosed with a glucose concentration > 3.3 mmol/L and PH > 7.3, and bacteria were found in pleural effusion culture or anti-infection treatment was effective. The diagnosis of tuberculous pleural effusion was based on the positive culture of mycobacterium tuberculosis or the diagnosis of caseous granuloma or pleural fluid adenosine deaminase > 40 µ/l with an improvement of the pleurisy after antituberculous treatment.

### Sample collection and determination of TuM2-PK and CEA levels

Pleural effusion was collected by percutaneous thoracic puncture and blood samples were simultaneously collected by vein for different tests. The extracted blood was coagulated under routine test for 15 min and centrifuged at 3000 revolutions per minute for 15 min to obtain serum. Under the same conditions, the pleural effusion was centrifuged and the cell-free supernatant was collected. Aliquots of serum and pleural fluid from this study were stored at − 70° C until analysis. TuM2-PK (Sche-Bo W Tech, Giessen, Germany) concentrations in serum and pleural fluid were measured by sandwich enzyme-linked immunosorbent assay (ELISA). CEA concentrations were measured by an electrochemiluminescence Kit (Roche Diagnostics, Beijing, China). All tests were done in two copies and diluted properly, and technicians ignored the clinical data.

### Statistical analysis

We used commercial SPSS 18.0 software for statistical analysis. Measurement data and numeration data were compared with t test and χ^2^ test, respectively. Diagnostic accuracy of tumor markers was compared by analyzing receiver operating characteristic (ROC). Results from patients with MPE were used to select cut-off values for sensitivity and specificity for all markers. Values of *P* < 0.05 were considered to indicate statistical significance.

## Results

### Clinical characteristics

Our study included 205 patients who were diagnosed with MPE and BPE. Among MPE patients, 91 (72.8%) were diagnosed with liquid cytology, 28 (22.4%) with blind pleural biopsy and 6 (4.8%) with thoracoscopic biopsy. The median age of the patients was 68 years (range 32–78 years), and 80 patients were male (64.0%). Most patients showed adenocarcinoma (64.0%). In patients with BPE, Bacteria were found in liquid smears and cultures of 20 patients, and chronic granuloma was found in pleural biopsy of 45 patients with BPE. In addition, a clear diagnosis was made and other possibilities were excluded. The demographic characteristics are shown in Table 1.

**Table 1:** Patient characteristics

Variables	MPE group	BPE group	*P* value
Subject, NO	125	80	
Age (year)	58.5 ± 11.8	59.2 ± 10.6	> 0.05
Male/Female	80/45	50/30	> 0.05
MPE			
Adenocarcinoma	80	ND	
Squamous cell carcinoma	25	ND	
Small cell lung carcinoma	20	ND	
BPE			
Tuberculosis	ND	50	
Parapneumonic	ND	30	
Diagnostic method			
Biochemistry	ND	15	
Bacteria	ND	20	
Cytology	91	ND	
Pleural biopsy	34	45	

MPE: malignant pleural effusion; BPE: benign pleural effusion; ND: no data

### Concentration of TuM2-PK and CEA in serum and pleural effusion

As shown in Table 2, patients with MPE presented higher serum and pleural effusion levels of TuM2-PK and CEA than those of BPE, the differences were statistically significant (*P* < 0.05). The P/S ratio was also significantly higher in MPE group than that of BPE group for TuM2-PK as well as CEA (*P* < 0.05).

**Table 2:** Concentration of TuM2-PK and CEA in serum and pleural effusion and P/S ratio in BPE and MPE

Marker	MPE	BPE	*P* value
TuM2-PK (µ/ml)			
Pleural effusion	56.38 ± 18.51	13.51 ± 1.65	0.001
Serum	33.25 ± 11.71	9.67 ± 2.87	0.001
P/S	1.79 ± 1.03	1.45 ± 0.46	0.034
CEA (ng/ml)			
Pleural effusion	37.28 ± 14.03	4.83 ± 0.48	0.001
Serum	21.57 ± 5.54	4.46 ± 0.63	0.001
P/S	1.78 ± 1.16	1.07 ± 0.08	0.021

MPE: malignant pleural effusion; BPE: benign pleural effusion

### Comparison of serum and pleural effusion TuM2-PK and CEA levels depending on pathology of MPE

**Fig. 1:**
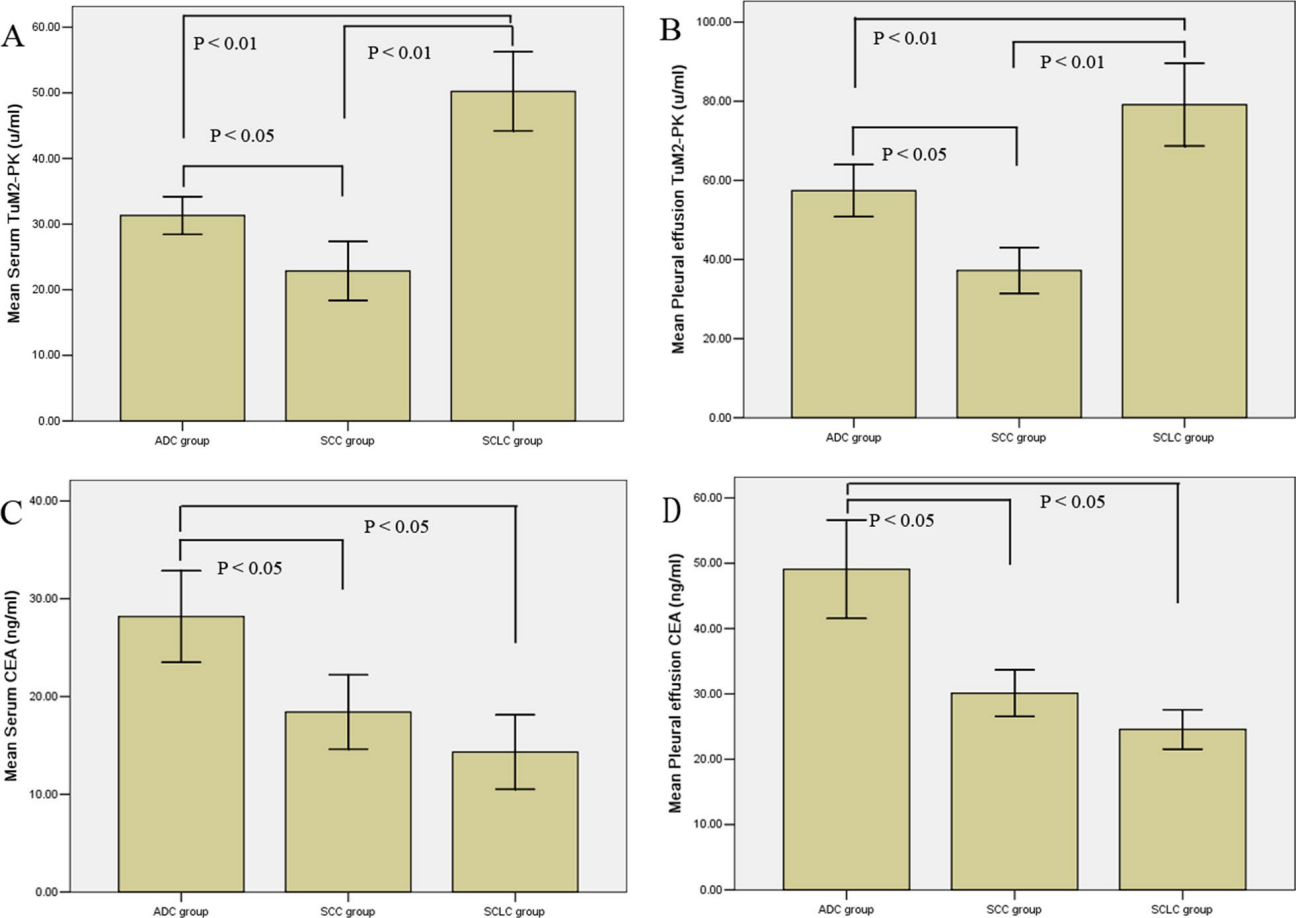
Comparison of serum and pleural effusion TuM2-PK (**A**, **B**) and CEA (**C**, **D**) levels depending on pathology of MPE

The levels of TuM2-PK in pleural effusion and serum of SCLC were significantly higher than that of lung ADC and SCC (*P* < 0.01), while the level of TuM2-PK in pleural effusion and serum of lung ADC was also higher than that of lung SCC (*P* < 0.05); CEA increased most significantly in lung ADC, and its level was significantly higher in lung SCC and SCLC (*P* < 0.05) (Fig. 1A–D).

### Diagnostic value of TuM2-PK and CEA in MPE patients

**Fig. 2:**
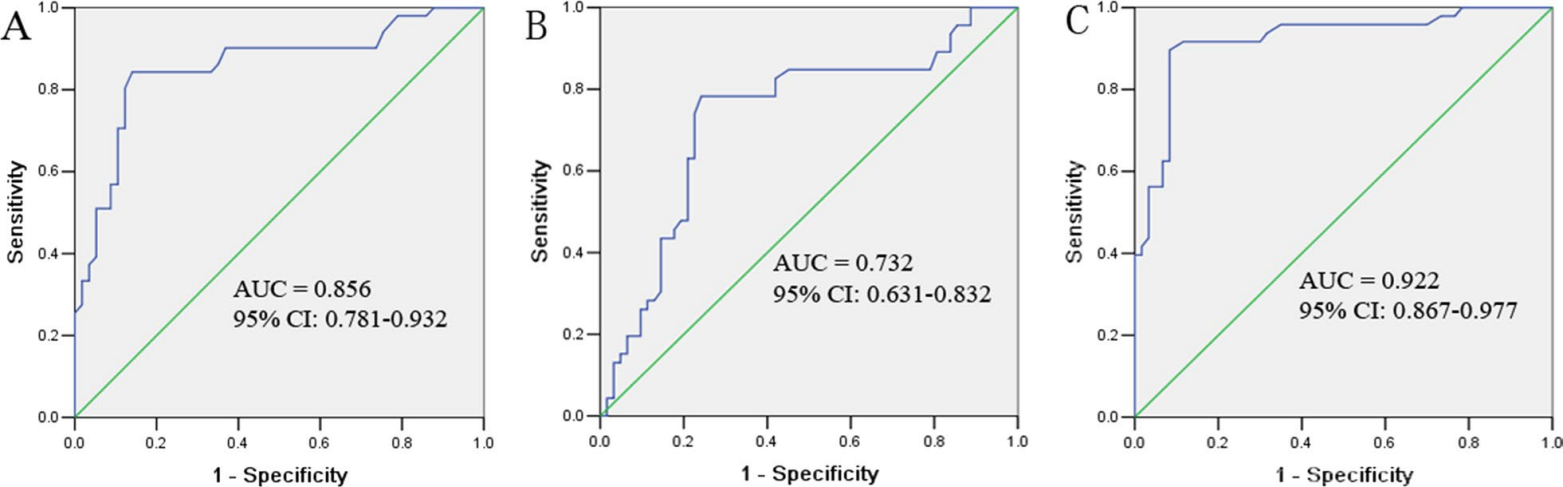
ROC curve in pleural effusion. TuM2-PK ROC curve (**A**), CEA ROC curve (**B**), TuM2-PK + CEA ROC curve (**C**). The AUC of pleural effusion TuM2-PK, CEA and TuM2-PK + CEA were 0.856, 0.732, and 0.922, respectively

ROC curves were plotted to determine the diagnostic efficiency of pleural effusion and serum TuM2-PK and CEA levels for MPE. ROC curve analysis showed that the area under the curve (AUC) of pleural effusion TuM2-PK, pleural effusion CEA and their combination were 0.856 (95% CI 0.781–0.932), 0.732 (95% CI 0.631–0.832) and 0.922 (95% CI 0.867–0.977), respectively. The AUC of serum TuM2-PK, serum CEA, and their combination were 0.816 (95% CI 0.729–0.903), 0.665 (95% CI 0.556–0.774) and 0.863 (95% CI 0.788–0.938), respectively. Therefore, the combination of the index is better than any single index (Table 3 and Fig. 2A–C).

**Table 3:** Measures of diagnostic accuracy for TuM2-PK and CEA assays in pleural effusion and serum in MPE

Marker	Cut-off		AUC (95% Cl)	Sensitivity (%)	Specificity (%)	Accuracy (%)	PPV (%)	NPV (%)
TuM2-PK								
Pleural effusion		28.67 u/ml	0.856 (0.781–0.932)	68.8	52.5	62.4	69.4	51.9
Serum		15.83 u/ml	0.816 (0.729–0.903)	60.8	58.8	60.0	69.7	49.0
CEA								
Pleural effusion		8.76 ng/ml	0.732 (0.631–0.832)	61.6	71.3	65.4	77.0	54.3
Serum		5.53 ng/ml	0.665 (0.556–0.774)	51.2	63.8	56.1	68.8	45.5
TuM2-PK + CEA								
Pleural effusion			0.922 (0.867–0.977)	91.2	83.8	88.3	89.8	85.9
Serum			0.863 (0.788–0.938)	80.8	71.3	77.1	81.5	70.4

AUC: area under the curve; PPV: positive predictive value; NPV: negative predictive value

Table 3 shows the diagnostic sensitivity, specificity, positive predictive value (PPV), negative predictive value (NPV), and accuracy of TM2-PK and CEA for diagnosing MPE. Cutoff points were determined by the maximum sum of sensitivity and specificity. The cutoff values of TuM2-PK and CEA in pleural effusion were 28.67 µ/l and 8.76 ng/ml, the sensitivity was 68.8% and 61.6%, the specificity was 52.5% and 71.3%, respectively. With a cut-off value of 15.83 µ/l and 5.53 ng/ml in serum TuM2-PK and CEA, the sensitivity was 60.8% and 51.2%, the specificity was 58.8% and 63.8%, respectively.

As shown in Table 3, the diagnostic efficiency of each marker was slightly higher in pleural effusion than in serum. The diagnostic efficiency of TuM2-PK alone was higher than CEA alone. The combined detection of TuM2-PK and CEA in pleural effusion or serum was superior to that of any single detection.

### Correlation between TuM2-PK and CEA in MPE patients

**Fig. 3:**
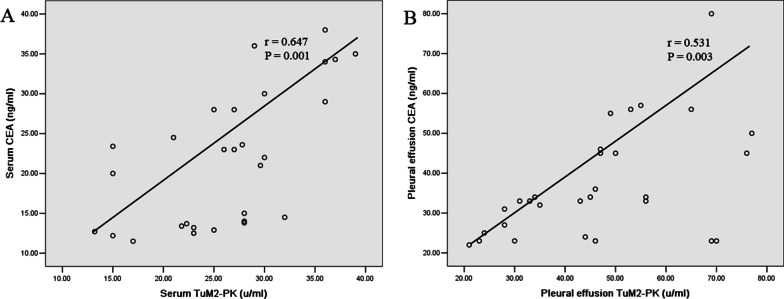
Correlation between TuM2-PK and CEA in MPE patients. There is a significant correlation between TuM2-PK and CEA levels in serum (**A**) and pleural effusion (**B**), respectively. (r = 0.647, *P* = 0.001; r = 0.531, *P* = 0.003)

There is a significant correlation between TuM2-PK and CEA levels in serum (Fig. 3A) and pleural effusion (Fig. 3B), respectively (r = 0.647, *P* = 0.001; r = 0.531, *P* = 0.003).

## Discussion

The conventional approach to diagnose the etiology of pleural effusions remains a challenge [16, 17]. The traditional pleural fluid cytology has been used to detect tumor cells in pleural fluid. However, sensitivity varies from 30 to 60% and the additional sensitivity provided by blindly obtained pleural needle biopsy specimens is minimal [18]. Although thoracoscopy is more sensitive to malignant cases, they are expensive and not available in all medical centers [19]. In recent years, with the development of tumor molecular biology, tumor markers have been widely used in the diagnosis of pleural effusion. We investigated TuM2-PK in the serum and pleural effusion of patients with pleural effusion caused by benign disease or lung cancer.

Previous studies have shown that TuM2-PK can be detected in body fluids, which may be due to the release of TuM2-PK during tumor necrosis or metastasis [20]. It has been reported that TuM2-PK was significantly increased in MPE, which can be used as an index to distinguish BPE and MPE [21]. The results showed that the levels of TuM2-PK in pleural effusion and serum of patients with MPE were significantly higher than those of patients with BPE, and increased most significantly in SCLC, which was significantly higher than those of lung ADC and SCC, while the levels of TuM2-PK in pleural effusion and serum of patients with ADC were also higher than those of patients with SCC, Therefore, TuM2-PK plays a certain role in the type and diagnosis of lung cancer. We found that the expression level of TuM2-PK in pleural effusion of patients with MPE was significantly higher than that in serum. TuM2-PK on the tumor surface was decomposed due to the increase of metalloproteinase activity, and penetrated into the pleural cavity. At the same time, TuM2-PK in the blood can also penetrate into the pleural cavity through pleural capillaries, so the concentration of TuM2-PK increases in MPE. Detection of TuM2-PK might be useful in differentiating BPE and MPE.

CEA has been widely used in the diagnosis of a variety of tumors. The concentration of CEA increases in the serum of 30–80% of lung cancer patients. The degree of increase is directly related to the number of cancer cells, and the positive rate for the diagnosis of lung ADC is the highest [22]. The results also confirmed that the CEA concentration and positive rate of lung ADC were significantly higher than those of SCC and SCLC, which was consistent with the literature report. There are also many reports on the use of CEA in the differential diagnosis of pleural effusion [23, 24]. The results of this study demonstrated that the CEA levels of serum and pleural effusion in MPE were significantly higher than those of BPE, which was basically consistent with the report [11–15].

We detected TuM2-PK and CEA in serum and pleural effusion of patients with MPE alone and in combination. It was found that the sensitivity and specificity of a single detection index in the diagnosis of MPE were less than 80%. Combined detection could increase the sensitivity and specificity. Therefore, the combined detection of TuM2-PK and CEA in serum and pleural effusion is helpful to the differential diagnosis of MPE and BPE, and combined detection is helpful to the pathological classification and clinical diagnosis of pleural effusion.

Several limitations of our study warrant discussion. First, we performed the study at a single centre with relatively small sample size. Second, the expression of TuM2-PK in serum of lung cancer patients was detected, but the expression of TuM2-PK in lung cancer tissues was not detected. Third, the specific mechanism of the relationship between TuM2-PK expression and MPE was lacking. Further perspective trial should be performed.

Our findings demonstrate that determining serum and pleural effusion levels of the markers TuM2-PK and CEA is useful in differentiating MPE and BPE. In comparison with either single determination of concentration in serum or pleural effusion, the combined detection of two markers was important in the diagnosis of lung cancer. In clinical practice, when the cytopathological examination of pleural effusion is negative, the simultaneous detection of TuM2-PK and CEA levels in serum and pleural effusion might help clinicians decide whether to obtain cytological or histological specimens by invasive methods to investigate the possible diagnosis of malignant tumors.

## Data Availability

All data generated or analysed during this study are included in this published article.
